# Child mortality, hypothalamic-pituitary-adrenal axis activity and cellular aging in mothers

**DOI:** 10.1371/journal.pone.0177869

**Published:** 2017-05-25

**Authors:** Cindy K. Barha, Katrina G. Salvante, Courtney W. Hanna, Samantha L. Wilson, Wendy P. Robinson, Rachel M. Altman, Pablo A. Nepomnaschy

**Affiliations:** 1 Maternal and Child Health Laboratory, Faculty of Health Sciences, Simon Fraser University, Burnaby, British Columbia, Canada; 2 Human Evolutionary Studies Program, Simon Fraser University, Burnaby, British Columbia, Canada; 3 Department of Medical Genetics, University of British Columbia, Vancouver, British Columbia, Canada; 4 Child and Family Research Institute, Vancouver, British Columbia, Canada; 5 Statistics and Actuarial Science, Simon Fraser University, Burnaby, British Columbia, Canada; University of Turku, FINLAND

## Abstract

Psychological challenges, including traumatic events, have been hypothesized to increase the age-related pace of biological aging. Here we test the hypothesis that psychological challenges can affect the pace of telomere attrition, a marker of cellular aging, using data from an ongoing longitudinal-cohort study of Kaqchikel Mayan women living in a population with a high frequency of child mortality, a traumatic life event. Specifically, we evaluate the associations between child mortality, maternal telomere length and the mothers’ hypothalamic-pituitary-adrenal axis (HPAA), or stress axis, activity. Child mortality data were collected in 2000 and 2013. HPAA activity was assessed by quantifying cortisol levels in first morning urinary specimens collected every other day for seven weeks in 2013. Telomere length (TL) was quantified using qPCR in 55 women from buccal specimens collected in 2013. Results: Shorter TL with increasing age was only observed in women who experienced child mortality (p = 0.015). Women with higher average basal cortisol (p = 0.007) and greater within-individual variation (standard deviation) in basal cortisol (p = 0.053) presented shorter TL. Non-parametric bootstrapping to estimate mediation effects suggests that HPAA activity mediates the effect of child mortality on TL. Our results are, thus, consistent with the hypothesis that traumatic events can influence cellular aging and that HPAA activity may play a mediatory role. Future large-scale longitudinal studies are necessary to confirm our results and further explore the role of the HPAA in cellular aging, as well as to advance our understanding of the underlying mechanisms involved.

## Introduction

Several lines of evidence support the hypothesis that exposure to stressors, including psychosocial, energetic, and health challenges, can influence the pace of biological aging [1–4]. The biological mechanisms underlying this relationship, however, are still being investigated. Stress has been hypothesized to exert its influence via several mechanisms, including the acceleration of cellular aging through an increase in the pace of telomere length (TL) attrition [5].

Located at the ends of chromosomes, telomeres are repetitive nucleotide sequences that protect chromosomal DNA from degradation. DNA degradation can result from incomplete replication (end-replication problem) [6,7], as well as damage from oxidative stress [8]. Once telomeres shorten past a critical length, cells undergo senescence [9]. While TL in human peripheral blood cells has been reported to decline with chronological age, the pace of attrition does not appear to be constant across the lifespan, with greater attrition observed both early and later in life [8,10,11]. It has been proposed that exposure to stress can accelerate this typical pattern of TL attrition [5]. Whether the effects of stress on TL attrition vary with age is not currently known.

The hypothalamic-pituitary-adrenal axis (HPAA) has been proposed as a possible physiological pathway mediating the relationship between stressful life events and the rate of TL attrition [12–17]. Stressors normally result in the activation of the HPAA which allows individuals to respond to the challenges at hand. Challenges stimulate the adrenal secretion of cortisol [18], resulting in the mobilization of metabolic energy to be used by the tissues involved in the response to the stressors at hand [19,20]. While adaptive, this process involves a biological cost. As metabolic resources are finite, energy mobilized to respond to a given challenge has to be reallocated away from regular metabolic tasks, such as somatic maintenance, including telomere maintenance and repair [21–23]. Thus, traumatic or frequent stress is hypothesized to lead to greater telomere attrition and, consequently, accelerated biological aging. Numerous cross-sectional studies (for example see: [24,25–28]) and a limited number of longitudinal studies [29,30] provide support for this hypothesis. A number of psychosocial stressors, including violence, financial hardship, divorce, unemployment, and caring for ill or elderly relatives, as well as other challenges, such as energetic stress, have consistently been associated with shorter telomeres (for review see: [5,31]). Yet, only a handful of studies have examined the role of HPAA activity as a modulator of TL in humans, and their results are equivocal [12–17]. While some of these studies reported negative associations between markers of HPAA activity, such as cortisol, and TL [12,13,15,17], others have not found any significant associations [14,16]. Therefore, whether the HPAA is directly involved in mediating the effects of stress on the pace of cellular aging is still a matter of much debate. Some of these inconsistencies between studies may be explained by methodological differences. These differences include the use of different biological matrices to assess cortisol levels (i.e., urine, saliva, blood), as well as variation in the number of specimens and the frequency with which they were collected, all of which can affect a study’s results. Specifically, HPAA activity varies dramatically within individuals as a result of various confounding factors, such as circadian rhythms, food consumption and physical activity [32,33]. We have previously argued that the use of first morning urinary cortisol allows for a tighter control of these confounders than cortisol quantification in other matrices such as saliva and blood [34]. Additionally, basal HPAA activity and responsivity varies markedly within and between individuals [35,36]. Thus, the single cortisol measurement frequently used in cross-sectional studies is not adequate to accurately assess HPAA activity [35]. Along the same lines, accurately assessing telomere attrition requires multiple TL measurements across time, which cross-sectional studies lack. Therefore, longitudinal cohort studies that include the repeated collection of biological specimens from participants can provide critical information regarding the role of HPAA activity as a mediator of the link between stress and TL attrition.

Taking into account these methodological issues, here we take advantage of an ongoing longitudinal-cohort study of Kaqchikel Mayan women to test the hypothesis that maternal exposure to child mortality, a traumatic life event, across a 13-year period is associated with age-related declines in TL. We also investigate whether HPAA activity plays a role in this relationship. We chose this stressor because child mortality is particularly high in this Mayan population (approximately 50% of women in this cohort experienced the death of one or more children). Consistent with the hypothesis that traumatic life events play a role in cellular aging, we predict that women who experienced the traumatic life event of child mortality will present shorter TL, and that this link will be significantly associated with the activity of their HPAA.

## Materials and methods

### Study population and participants

The analyses are based on a cohort of 107 Kaqchikel Mayan women initially recruited in the year 2000 for the Society, Environment and Reproduction (SER) study [37,38]. SER is a naturalistic, longitudinal study focused on the relationship between “naturally occurring” stress and women’s reproductive function. All participants are Kaqchikel Maya with at least 5 generations of traceable ancestors, which reduces genetic variability within our sample. Variation in terms of lifestyle (i.e., diet, physical activity, education, and socio-economic status) is lower in our population than that usually found among women living in urban communities of industrialized populations. This homogeneity reduces the potential confounding effects of cultural and lifestyle factors. Women in our study population did not smoke. This is particularly important, as previous studies have shown a negative influence of cigarette smoking on telomere length [26].

Of the 107 SER participants originally recruited in the year 2000, 94 volunteered to participate in the current study and provided urinary biospecimens and a buccal epithelial cell sample, as well as a bioelectrical impedance measurement (to gauge percent body fat [39]) in the year 2013. Of the 94 women, 19 were excluded from our sample because critical demographic information was missing for 2013 (e.g., no information on parity or child mortality) [40]. These exclusions resulted in a sample size of 75 women. For the present study, women who were pregnant or in postpartum amenorrhea in 2013 (n = 20) were further excluded from analyses because HPAA activity has been shown to differ between these reproductive stages and cycling women [41–43]. This resulted in a final sample of 55 women. The average age of the 55 participants in 2013 was 39.8 ± 5.8 years (average ± sample SD; range: 29–53 years) (Table 1).

**Table 1:** Characteristics of the study population. Values are average ± sample SD (range).

Traits	Study Population (n = 55)	Did not experience the loss of a child (n = 30)	Experienced the loss of a child (n = 25)
Age in 2013 (years)	39.8 ± 5.8 (29–53)	38.4 ± 6.0* (29–53)	41.5 ± 5.0* (31–53)
Total number of children	5.6 ± 2.1 (1–10)	5.3 ± 1.7 (3–10)	5.9 ± 2.4 (1–10)
Average first morning urinary cortisol (log-10)	1.32 ± 0.18 (0.84–1.73)	1.31 ± 0.18 (1.04–1.62)	1.33 ± 0.18 (0.84–1.73)
SD first morning urinary cortisol (log-10)	0.22 ± 0.07 (0.09–0.39)	0.22 ± 0.08 (0.09–0.39)	0.23 ± 0.07 (0.11–0.38)

* *p* < 0.05 for comparison between groups.

### Procedures

#### Ethics

Data collection in the year 2000 was approved by the University of Michigan’s Institutional Review Board. Data collection in 2013 and data analyses were approved by Simon Fraser University’s Ethics Review Board and the University of British Columbia’s Clinical Ethics Review Board. Informed consent was obtained orally from illiterate individuals and in writing from literate ones. In all cases the consent document was read in Kaqchikel Mayan, the local language, by a female research assistant to each prospective participant and signed by the participants with a cross, finger print or name initials, according to their individual preferences. The University of Michigan’s Institutional Review Board approved the 2000 consent procedure and study protocols, and Simon Fraser University’s Ethics Review Board approved the ones used in 2013. The authors assert that all procedures contributing to this work comply with the ethical standards of the relevant national and institutional committees on human experimentation and with the Helsinki Declaration of 1975, as revised in 2008.

#### Assessment of exposure to child mortality

Child mortality was assessed via comparison of demographic interviews administered in 2000 and 2013. Trained local, bilingual female research assistants administered the interview in Kaqchikel (one of two local languages), and recorded all of the answers by hand. This interview included questions about age, number of children born to a woman, family income and child mortality. Child mortality included all post-natal deaths, but not miscarriages or stillbirths as we did not have complete information regarding these two types of child mortality. Of the children who died, most did so after 6 months of age, mainly from infection-related illnesses, which induced diarrhea, vomiting and fever. Based on the demographic information, we created a binomial variable to identify women who experienced the death of at least one child between 2000 and 2013 (n = 25) and those who did not (n = 30). Two of the women in our sample, one in each group, were post-menopausal.

#### Telomere length assessment

TL was measured in cells recovered from buccal epithelial specimens collected in 2013. A buccal specimen was collected from each participant using an SK-1 Isohelix Buccal Swab (Cat. No: SK-1S) by rubbing the swab firmly against the inside of the right cheek for 1 minute. Swabs were then stored with an Isohelix Dri-Capsule (Cat. No: SGC-50), which is designed to ensure long-term buccal DNA stability at room temperature for over three years [44,45]. Buccal samples were stored at room temperature in a dark, dry cabinet for approximately 2 months, then transported at room temperature from the field site to our laboratory where they were similarly stored for 6 months until analysis.

Genomic DNA was extracted from buccal samples using the DDK DNA Isolation Kit (Isohelix Ltd, Kent, UK). DNA quality was checked by running extracted genomic DNA from a subsample of buccal samples for 1 hour on a 0.8% agarose gel with a Hind III ladder. DNA from buccal samples was found to be of sufficient quality, with high molecular weight DNA observed in all tested samples. DNA samples were quantitated using the NanoDrop 2000 UV-Vis Spectrophotometer (Thermo Scientific, Wilmington, USA) and diluted to 10ng/uL in 10mM Tris, 0.1mM EDTA buffer. Quantitative PCR (qPCR) was used to measure average relative TL (T/S ratio) as previously described [46]. Briefly, quantification of the amplification of telomeric repeats relative to a single-copy gene, *36B4*, was performed using SYBR green. The 36B4 gene, which encodes acidic ribosomal phosphoprotein PO, is located on chromosome 12. The telomere primers were designed to allow for amplification of the smallest possible amplicon (76bp). Consequently, the qPCR amplification (C_T_ value) and the amount of primer binding sites in the genomic DNA template were proportional [47]. The primer sequences (written 5′→3′) were: tel 1, GGTTTTTGAGGGTGAGGGTGAGGGTGAGGGTGAGGGT; tel 2, TCCCGACTATCCCTATCCCTATCCCTATCCCTATCCCTA; 36B4u, CAGCAAGTGGGAAGGTGTAATCC; 36B4d, CCCATTCTATCATCAACGGGTACAA. Standard curves and control samples were generated from whole blood DNA [46].

Sample identifiers were blinded to the experimenter and, thus, sample groups were randomly distributed among the runs. During preparation, samples were distributed in a non-replicated order across each plate, with each sample replicate always run on an independent plate. To monitor reproducibility, samples were run in triplicate within each 96-well plate, with at least two independent replicates. For the current study samples, 28 sets of 96-well T and S plates were completed. Across the 28 sets of plates, the average R^2^ was 0.995 (± 0.003). C_T_ values were excluded if the standard deviation among triplicates was greater than 0.20. Furthermore, if the coefficient of variation between the two independent replicates was greater than 0.15, two additional independent replicates were performed. Independent replicates were then averaged to generate an average relative TL for each sample (T/S ratio), the ratio of the number of telomere repeat copies (T) to the number of *36B4* gene copies (S). As the efficiencies of the T plates (77.8% ± 6.4%) were consistently lower than those of the corresponding S plates (85.6% ± 3.4%), we used the Pfaffl method [48] to calculate T/S ratios, which takes into account this variation in plate efficiency. The average intra-plate coefficient of variation for C_T_ was 0.33% (±0.07%) and for T/S values was 6.28% (±0.97%). The average inter-plate coefficient of variation was 5.23% (±3.56%).

T/S ratio and the average TL of a given sample have previously been shown to be proportional [47]. While the ‘gold standard’ for TL measurement is Southern blot of the terminal restriction fragment (TRF), the qPCR-based method has several advantages, including the ability to use smaller amounts of DNA and its high reproducibility, which have led to a recent increase in its use [49–51]. The reproducibility of our qPCR methodology was previously validated by blind comparison of measurements obtained using qPCR and the flow fluorescence in situ hybridization (flow-FISH) technique (r = 0.96; [46]).

#### Assessment of hypothalamic-pituitary-adrenal axis activity

Basal HPAA activity was determined by quantifying free unconjugated cortisol levels in first morning urinary (FMU) biospecimens collected every other day over a period of seven weeks in 2013. FMU cortisol provides an integrative measure of basal HPAA activity and is less likely to be affected by diurnal confounders than cortisol quantified in other matrices such as serum and saliva [34,52]. Participants provided, on average, 19 ± 1.90 FMU samples each (average ± sample SD; range: 13–22). This sampling collection protocol permitted us to determine an average level of FMU cortisol for each individual and monitor day-to-day variation in urinary free cortisol levels within- and among-individuals [35]. Upon waking, participants collected their first urinary voids into sterile, inert plastic containers provided by our research team the night before the collection day. Within 4 hours of being produced, specimens were aliquoted into 2 ml cryovials, and stored at -10°C in the field. Within six months of their collection, samples were shipped on dry ice to our laboratory and archived at -80°C until analysis. FMU cortisol was quantified using a previously validated enzyme immunoassay (EIA) array (Quansys Biosciences, Logan, UT) [53], with a lower limit of detection of 0.5ng/ml. All samples were run in duplicate and intra- and inter-assay coefficients of variation were 6.7% and 12.0%, respectively. The cortisol concentration of each sample was corrected for specific gravity using refractometry to adjust for variation in hydration state [54,55].

#### Statistical analyses

We fit a linear multivariate regression model using JMP (version 12, SAS Institute) to describe buccal TL as a function of the predictor variables. Specifically, we included child mortality, age, the interaction between child mortality and age, average FMU cortisol and intra-woman standard deviation (SD) of daily FMU cortisol (markers of HPAA activity) as predictors. Whereas average FMU cortisol is reflective of basal HPAA activity, SD cortisol reflects within-woman variability in HPAA activity among days. Higher SD in FMU cortisol may indicate that an individual has a more sensitive or reactive HPAA, or possibly, that an individual has been exposed to more challenges (e.g., psychosocial stress, infection, energetic stress, etc.). All FMU cortisol concentrations were log-10 transformed to normalize data distribution.

Previously we showed that increased parity is associated with reduced telomere shortening in women in our study population [40]. To account for this effect of parity, we included total number of children born to a woman as a covariate in our model. We also included percent body fat as a covariate in the model to account for mothers’ body composition as a potential confounding factor. Percent body fat was not a significant covariate (p > 0.424). As excluding percent body fat from the model did not change the estimates or significance for the other parameters, we excluded it to avoid over-fitting [56]. Thus, our final linear multivariate regression model was: buccal TL ~ age + child mortality group + age*child mortality group + average FMU cortisol + within-woman SD in FMU cortisol, with number of children born to a woman entered as a covariate. This final model included our effects of interest while adjusting for known, available confounders. The significance level was set at 0.05.

**Fig 1:**
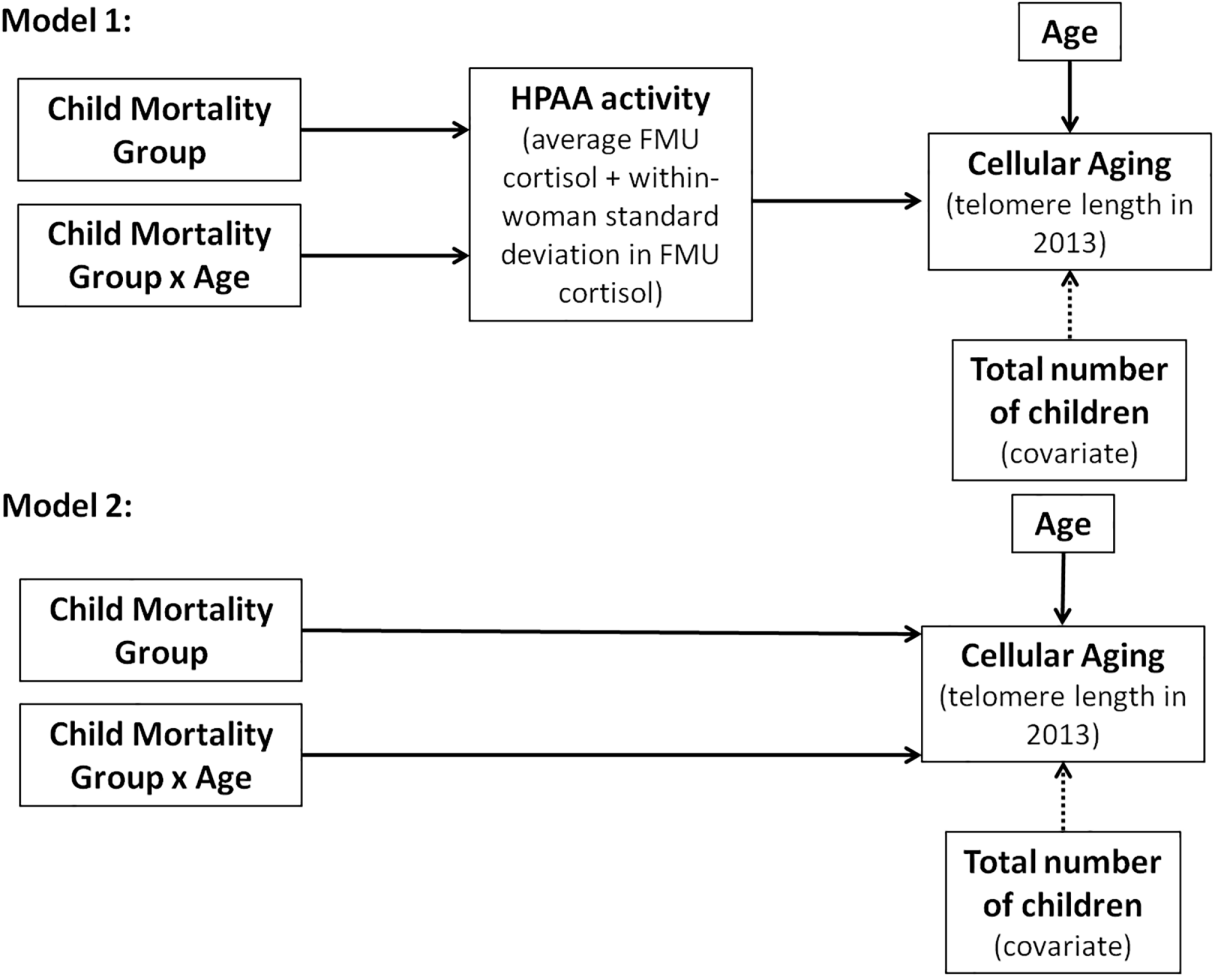
Mediation model. The two models compared to examine whether HPAA activity mediates the main effect of child mortality group and the interaction effect of child mortality and age on telomere length, including total number of children as a covariate (dotted lines).

To evaluate whether HPAA activity (average FMU cortisol and within-woman SD in FMU cortisol) mediated the associations between 1) child mortality variable and buccal TL and 2) the child mortality x age interaction and buccal TL, we used a non-parametric, bias-corrected and accelerated (BC_a_) bootstrapping procedure in R (Version 3.3.1, 2016 R Development Core Team, Vienna, Austria) to estimate the mediation effect [57–60]. The bootstrapping procedure has been recommended for regression-based mediation because mediation effect estimates are generally not normally distributed unless sample size is very large [61]. Briefly, for each of 10,000 bootstrapped samples, two models were fit: the first including all of the variables in our final linear multivariate regression model (Model 1, Fig 1), and the second excluding average FMU cortisol and within-woman SD in FMU cortisol (Model 2, Fig 1). The differences between the estimated main effects of child mortality group in the two models and between the estimated effects of the child mortality group by age interaction in the two models were computed. These differences, if significantly different from 0, would suggest a mediatory role for HPAA in the associations between buccal TL and experiencing child mortality and between buccal TL and the interaction between experiencing child mortality and age [59].

## Results

The average age of the 55 participants in 2013 was 39.8 ± 5.8 years (average ± sample SD; range: 29–53 years) (Table 1). Women in the group who experienced child mortality were, on average 3 years older than those in the group who did not experience this life event (*p* = 0.040) (Table 1). Average log-10 transformed FMU cortisol was 1.32 ± 0.18 across seven weeks in 2013, and the average within-woman standard deviation (SD) of daily log-10 transformed FMU cortisol across seven weeks in 2013 was 0.22 ± 0.07 (Table 1). The 55 participants had an average of 5.6 ± 2.1 children (Table 1). Average and SD FMUC cortisol and total number of children were not significantly different between the two groups (all p’s > 0.05).

**Fig 2:**
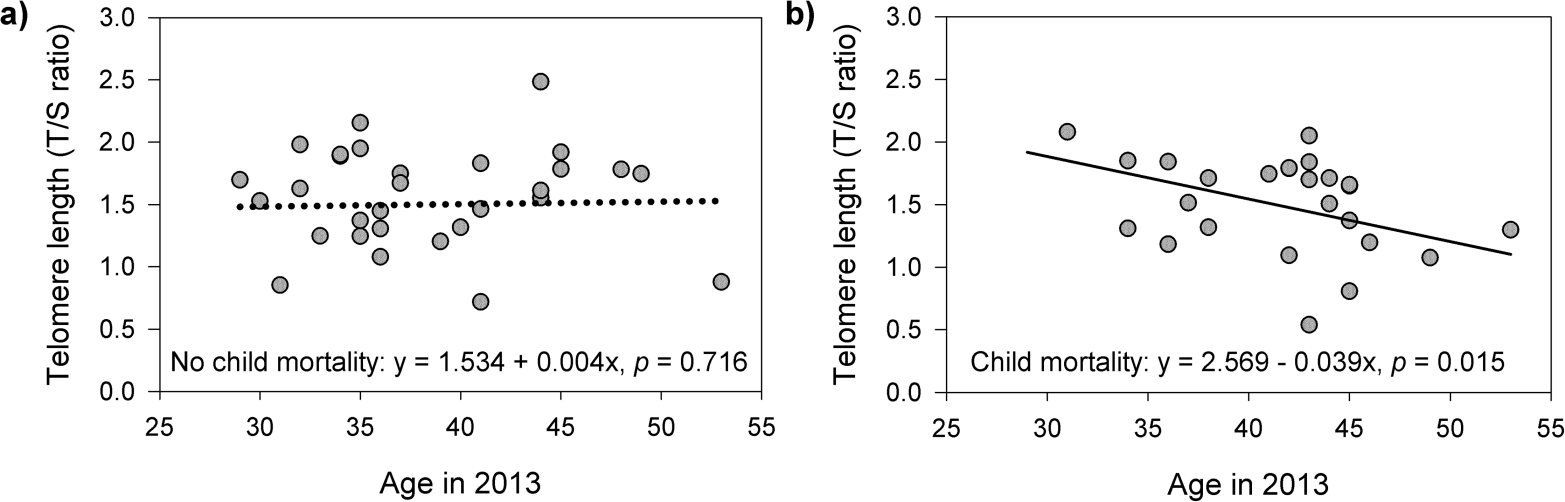
Relationships between buccal telomere length, child mortality and maternal age. a) Increasing chronological age was significantly associated with shorter buccal telomere lengths in women who experienced the death of one or more children (slope = -0.039, *p* = 0.015), b) but not in women who had not experienced child mortality (slope = 0.004, *p* = 0.716), after adjusting for all other predictors and covariates. The lines in each graph represent the estimated relationship between buccal telomere length and age for a woman with average values for all of the other predictors and covariates in the model.

**Fig 3:**
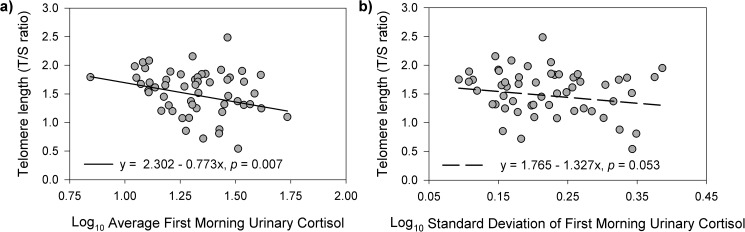
Relationships between buccal telomere length and HPAA activity. Regardless of child mortality exposure, shorter buccal telomere lengths were also a) significantly associated with higher first morning urinary cortisol levels (slope = -0.773, *p* = 0.007) and b) marginally associated with higher within-woman variation (standard deviation) in first morning urinary cortisol levels (slope = -1.327, *p* = 0.053), after adjusting for all other predictors and covariates. The lines in each graph represent the estimated relationship between buccal telomere length and average first morning urinary cortisol and within-woman standard deviation in first morning urinary cortisol levels, respectively, for a woman with average values for all of the other predictors and covariates in the model.

Our final linear multivariate regression model explained 26% of the variation in buccal TL (p = 0.018) (Table 2). The relationship between maternal age and buccal TL was significantly associated with the experience of losing a child (age x child mortality interaction: *p* = 0.025). In women who did not experience child mortality, buccal TL was not significantly associated with age (B = 0.004, SE = 0.011, *p* = 0.716; Fig 2A), after adjusting for total number of children. In contrast, in women who experienced child mortality, buccal TL was negatively associated with age (B = -0.039, SE = 0.015, *p* = 0.015; Fig 2B), after adjusting for total number of children. Average FMU cortisol was negatively associated with buccal TL (B = -0.773, SE = 0.276, *p* = 0.007), after adjusting for total number of children. A similar negative trend was observed for SD of FMU cortisol (B = -1.327, SE = 0.667, *p* = 0.053). In other words, women with higher and more variable FMU cortisol levels exhibited shorter buccal TL (Fig 3A and 3B, respectively).

**Table 2:** The relationships between buccal telomere length and child mortality, age, and basal HPAA activity, adjusted for number of children born to each woman. Final linear multivariate regression model: buccal TL ~ age + child mortality group + age:child mortality group + average FMU cortisol + within-woman SD in FMU cortisol + number of children born to a woman (covariate) (R^2^ = 0.26, p = 0.018).

Variable	B	Std Error	p-value
Intercept	2.400	0.515	< 0.0001
**Covariate**			
Number of children born to a woman	0.057	0.026	0.033
**Predictor Variables**			
^a^ Age in 2013	0.004	0.012	0.716
Child mortality	1.689	0.752	0.029
^b^ Age in 2013 * Child mortality	-0.043	0.018	0.025
Average log_10_ first morning urinary cortisol	-0.773	0.276	0.007
Standard deviation log_10_ first morning urinary cortisol	-1.327	0.668	0.053

^a^ Effect of age on buccal TL in women who did not experience child mortality is 0.004

^b^ Effect of age on buccal TL in women who experienced child mortality is 0.004–0.043 = -0.039

In evaluating whether HPAA activity (average and SD FMU cortisol) mediated the associations between the child mortality and buccal TL, we found that 95% of the bootstrap estimated differences in the main effect of child mortality fell below 0, while 95% of the bootstrap estimated differences in the child mortality by age interaction effect fell above 0. Therefore, the estimates for both the main effect of experiencing child mortality and the child mortality by age interaction differed significantly between the model that included the HPAA activity variables and the model that excluded these variables, suggesting that HPAA activity may act as a mediator of the effect of child mortality on TL.

## Discussion

The rate of age-associated decline in TL is often assumed to be relatively consistent among individuals across time [62]. Nonetheless, exposure to various insults, including social, environmental and physiological challenges, have been hypothesized to accelerate this process [5,24,25,27,28,31,63–66]. The evidence supporting this hypothesis, however, has been mixed, with some studies supporting this association [12,13,15,17], while other studies have not [14,16]. Furthermore, the mechanisms involved in the putative effects of stress on the pace of cellular aging are still being investigated. Our results show a link between experiencing a traumatic challenge–child death–and maternal age-related TL loss, which is consistent with the original hypothesis regarding the effects of stress on the pace of cellular aging. Our analyses are consistent with the prediction that HPAA activity is involved in the mechanism mediating the association between stress and cellular aging.

Interestingly, the age-associated decline in TL was only observed in women who experienced child mortality. The lack of an obvious association between age and TL in those who did not experience this traumatic event may be explained by the limited age range of the women in our study (29 to 53 at follow-up in 2013). While TL is generally expected to decrease with age (for review see: [67,68]), within the age range of the women in our sample, this decline was anticipated to be minimal. Indeed, in human peripheral blood cells, TL attrition follows a biphasic pattern, accelerated in early childhood and in old age, with a possible period of relative quiescence during adulthood [8]. The age range of the women in our sample during the period that we observed them (average age in 2000 = 25.37 years; average age in 2013 = 38.37 years) falls within the period of hypothesized relative quiescence. This overlap may explain why the women who did not suffer child mortality did not exhibit a statistically significant age-associated TL decline. The maintenance of TL during this period of adulthood in women may be explained by protective factors associated with women’s reproductive physiology and pregnancy that may mitigate TL shortening. Ovarian cycling is accompanied by regular increases in circulating levels of estradiol. Estradiol is known to promote telomerase activity and reduces oxidative stress levels [69–72], two factors that can protect TL. Pregnancy is accompanied by even higher and more prolonged increases in circulating estradiol levels. Thus, parity may exert additional protective effects on TL. Furthermore, pregnancy has been argued to confer protection against the age-related decline in the regenerative capacity of various maternal tissues [73]. These effects have been observed in a variety of tissues, including the liver, central nervous system, and heart, in a number of vertebrate species, ranging from mice to humans [73]. Several mechanisms have been proposed as potential mediators of these effects including the parabiotic-like effects of microchimeric fetal cells on the regenerative capacity of maternal tissues, as well as those of pregnancy-related maternal hormones, like estradiol and prolactin [73]. In mice models, for example, elevated prolactin induces an increase in neural precursor cells leading to an increase in newly generated oligodendrocytes, which are involved in the remyelination of axons in experimentally demyelinated animals [73]. These protective effects associated with reproduction, could be linked to, and perhaps help explain, the quiescence period in TL attrition in women during their reproductive years, particularly in high fertility populations such as the one we study. Consistent with this hypothesis, in our study population the total number of children born to a woman is positively correlated with TL [40]. The existence and extent of the protective effects of pregnancy on aging and the period of relative quiescence in TL attrition during women’s reproductive years is still being investigated and debated. Future research is needed to further examine these effects as well as any other factors that may affect the pace of TL attrition in women across different life stages (e.g., pre-puberty vs. adulthood vs. post-menopause).

The negative association we observed between child mortality and age-related variation in maternal TL during a woman’s reproductive years is consistent with the hypothesis that the putative protective effects of maternity on aging [40,73] can be overcome by the stress elicited by exposure to traumatic events. Our results parallel those of other studies showing that traumatic events are linked to the pace of telomere attrition across the human lifespan. Shalev et al. [30], for example, showed that exposure to violent events during childhood exacerbated telomere attrition in children between the ages of five and ten. In post-menopausal women, major life stressors, such as loss of household, major financial difficulties or death of a family member or friend, were associated with greater telomere attrition over a one-year period [29]. Our results extend these findings, showing that traumatic events are associated with shorter TL even in the life stage during which TL seems to be the most stable.

### A role for the HPAA?

To our knowledge, this is the first study to use a longitudinal design to evaluate the role of HPAA activity as a potential mechanism through which traumatic life events may affect the age-associated variation in TL. Consistent with our prediction, our results showed that women with higher FMU cortisol levels presented shorter TL. In terms of mediating mechanisms, there is evidence suggesting that exposure to high circulating cortisol leads to increased oxidative stress [74], which preferentially damages the telomere region of the chromosome [64,75]. Additionally, previous work has shown that *in vitro* exposure of human T lymphocytes to cortisol can reduce the activity of telomerase [76], an enzyme that protects telomeres from shortening by adding back telomeric DNA [77]. Consistent with these observations, Epel and colleagues found that caregivers, who have greater levels of perceived stress, showed reduced telomerase activity at baseline and in reaction to an acute stressor compared to a non-caregiver group [78]. Similarly, higher fasting morning serum cortisol levels have been reported to be associated with reduced telomerase activity in a randomized mindfulness intervention study [79]. Another potential mechanism through which cortisol may influence telomere attrition involves increased division and proliferation of cells in tissues with high levels of cell turnover [80–82]. Rapid cell turnover can lead to increased TL shortening through the end-replication problem [6,7]. Our observation that HPAA activity was associated with TL may have been facilitated by our use of buccal epithelial cells to assess TL, because these cells have a rapid turnover rate. Together, our results and those of previous studies discussed above are consistent with the hypothesis that high levels of HPAA activation may influence cellular aging via oxidative stress and telomerase pathways and increased cell turnover.

Of note, we observed a trend towards a negative correlation between intra- individual variation (SD) in basal FMU cortisol and TL while accounting for variation in total number of children. The functional significance of intra-individual variability in HPAA activity has not received due attention in the literature. This variability has been argued to provide a measure of how often the HPAA is activated (i.e., the level of physiologic stress to which an individual is exposed) and may also reflect past exposure to stress [35,83]. Thus, the observed trend suggesting a negative link between within-individual basal cortisol SD and TL is also consistent with the hypothesis that life challenges may affect cellular aging through changes in HPAA activity.

The estimated mediating effects of average and SD FMU cortisol on the association between experiencing child mortality and TL were not large. This may be due to a variety of factors, including our relatively small sample size and our choice of HPAA activity measurement. In this study, we evaluated HPAA activity using FMU cortisol, an integrated measure of cortisol secretion across the night. Alternative HPAA measures, including the pattern of cortisol reactivity to and recovery from a stress challenge or diurnal cortisol profiles may provide more information as to the role of HPAA activity in cellular aging. Future studies that evaluate other aspects of HPAA activity and their potential to mediate the relationships between stressful life events and cellular aging are needed.

### Limitations

Inherent to all observational studies, we cannot rule out the possibility that the relationship observed between child mortality and TL in mothers could be explained by unmeasured factors not considered in our analyses. Socioeconomic status could potentially influence both maternal TL and child mortality risk [84]. At the time of the study, however, our rural, indigenous study population was socioeconomically quite homogenous compared to industrialized communities. Thus, it is unlikely that this variable alone could explain our results.

Mothers who experienced child mortality were, on average, 3 years older than those who did not. This difference is to be expected as the probability of losing a child should be directly proportional to a woman’s age (in this population the more time goes by, the more children a woman is likely to have and the greater the chances that one of her children may die). Thus, even though the age ranges were the same between the two groups, the group of women who experienced child mortality had fewer younger women than the group of women who didn’t. It could be argued that the link we observed between child mortality and TL may be attributed to this difference in average age. However, as age was included in our model as a predictor variable, the effect of age is accounted for in our analyses.

Maternal body condition could also potentially confound the relationship between child mortality and TL. However, when percent body fat was included in our initial model as a covariate to account for body condition, it was not associated with TL, nor did its exclusion from the model change the estimates or significance of any of the other model parameters. Other measures of body condition that were not measured, however, could still act as potential confounding factors. Further, as some of our qPCR reaction efficiencies were slightly lower than other studies that used qPCR to quantify TL [85,86], the results should be taken with caution.

We were not able to determine the specific date at which the traumatic event (child death) occurred for each woman, limiting our ability to assess the temporal influence of stress on TL shortening. This may be of particular interest when considering previous work suggesting that the time between exposure to stressful life events and TL measurement may have an impact on the strength of the association between the two variables [26]. More recent events may also have stronger effects on basal HPAA activity than past events. Future studies should also endeavour to assess the temporal nature of traumatic stressful events as well as HPAA activity closer to the time of the event. Despite these limitations, the present study offers novel information that contributes to our understanding of the role that life events may have on cellular aging. Larger epidemiological studies of women of different ethnicities will be necessary to replicate our analyses and confirm our results.

### Conclusions

Our results are consistent with the hypothesis that traumatic life events can affect the pace of cellular aging in women of reproductive age, and suggest that maternal HPAA activity may mediate this association. Additional large-scale longitudinal studies are necessary to confirm and further explore the effects of other traumatic life events on cellular aging and the role of the HPAA. These studies should incorporate the collection of multiple biological markers of stress physiology and cellular aging to further elucidate the biological mechanisms involved.
